# A diversity-generating retroelement encoded by a globally ubiquitous *Bacteroides* phage

**DOI:** 10.1186/s40168-018-0573-6

**Published:** 2018-10-23

**Authors:** Sean Benler, Ana Georgina Cobián-Güemes, Katelyn McNair, Shr-Hau Hung, Kyle Levi, Rob Edwards, Forest Rohwer

**Affiliations:** 1 Department of Biology, San Diego State University, 5500 Campanile Drive, San Diego, CA 92182 USA; 2 Department of Computer Science, San Diego State University, 5500 Campanile Drive, San Diego, CA 92182 USA

**Keywords:** Bacteroides, Diversity-generating retroelements, Prophage, Viromes

## Abstract

**Background:**

Diversity-generating retroelements (DGRs) are genetic cassettes that selectively mutate target genes to produce hypervariable proteins. First characterized in *Bordetella* bacteriophage BPP-1, the DGR creates a hypervariable phage tail fiber that enables host tropism switching. Subsequent surveys for DGRs conclude that the majority identified to date are bacterial or archaeal in origin. This work examines bacteriophage and bacterial genomes for novel phage-encoded DGRs.

**Results:**

This survey discovered 92 DGRs that were only found in phages exhibiting a temperate lifestyle. The majority of phage-encoded DGRs were identified as prophages in bacterial hosts from the phyla Bacteroidetes*,* Proteobacteria, and Firmicutes. Sequence reads from these previously unidentified prophages were present in viral metagenomes (viromes), indicating these prophages can produce functional viruses. Five phages possessed hypervariable proteins with structural similarity to the tail fiber of BPP-1, whereas the functions of the remaining DGR target proteins were unknown. A novel temperate phage that harbors a DGR cassette targeting a protein of unknown function was induced from *Bacteroides dorei*. This phage, here named *Bacteroides dorei* Hankyphage, lysogenizes 13 different *Bacteroides* species and was present in 34% and 21% of whole-community metagenomes and human-associated viromes, respectively.

**Conclusions:**

Here, the number of known DGR-containing phages is increased from four to 92. All of these phages exhibit a temperate lifestyle, including a cosmopolitan human-associated phage. Targeted hypervariation by temperate phages may be a ubiquitous mechanism underlying phage-bacteria interaction in the human microbiome.

**Electronic supplementary material:**

The online version of this article (10.1186/s40168-018-0573-6) contains supplementary material, which is available to authorized users.

## Background

Phages encode genes selectively mutagenized by diversity-generating retroelements (DGRs) [1–3]. DGRs are genetic cassettes that introduce DNA sequence variation through a unique targeted mutagenesis mechanism [4]. The mechanism of DGR function is best understood in *Bordetella* phage BPP-1. BPP-1 possesses a 134 bp variable repeat (VR) within the gene encoding the phage tail fiber. Downstream of VR is a second, invariant copy designated the template repeat (TR). Nearby the TR/VR pair are genes encoding an “accessory variability determinant” and a reverse transcriptase (RT). These two proteins generate an error-prone cDNA from the TR, followed by stable incorporation of the mutagenized cDNA into the phage tail fiber gene [4, 5]. This process, termed “mutagenic retrohoming,” yields a VR that is distinct from the TR exclusively at adenine bases. As a result, the BPP-1 tail fiber that mediates adsorption to bacterial host receptors is hypervariable, enabling tropism switching on host *Bordetella* species [4, 6, 7].

Since the initial discovery in BPP-1, the majority of DGRs identified to date are considered bacterial and archaeal in origin [6, 8–11]. A recent survey found that 40% of DGRs are flanked by genes with sequence similarity to phages [12]. However, it is currently an open question whether these cassettes are encoded by functional prophages or inactive remnants.

Here, a survey of phage genomes found that DGRs are only encoded by phages exhibiting a temperate lifestyle. Investigation of bacterial genomes found that DGRs previously considered to be bacterial in origin are encoded by functional prophages. These phage DGRs diversify proteins with unknown functions, though most possessed a C-type lectin domain. A single temperate phage, Hankyphage, was found to harbor a DGR cassette and lysogenize at least 13 different species of the genus *Bacteroides*. Hankyphage was present in whole-community metagenomes and viromes generated from microbial samples collected around the globe.

## Results

### DGRs found in three temperate phages

A comprehensive survey of phage genomes was conducted to identify novel phage-encoded DGRs. Searching publicly available genomes from isolated phages (*n* = 4203, NCBI RefSeq and PhagesDB databases) identified six phages that possessed a reverse-transcriptase (RT). Three of these six possessed features characteristic of a DGR cassette: an adjacent variable repeat (VR)/template repeat (TR) pair containing > 7 adenine mismatches (Additional file 1). The paradigmatic DGR-harboring phage BPP-1 was identified, as well as two marine *Vibrio* phages. The VR of the BPP-1 DGR lay within the phage tail fiber gene, while the VRs of the two marine *Vibrio* phages were encoded within genes of unknown function. All three genes harbored a C-type lectin domain, consistent with previous structural analyses of DGR target proteins [13].

### DGRs encoded by novel temperate phages

To more exhaustively search for DGRs in temperate phages, the survey was expanded to include temperate phages integrated into genomes as prophages. A database of 31,946 predicted prophage-containing regions generated by PhiSpy from 11,278 bacterial genomes was scanned for DGRs [14]. PhiSpy utilizes both sequence homology-dependent and independent approaches to predict regions harboring an integrated phage [15]. In total, 170 regions harbored a DGR cassette (Additional file 2). Previous surveys identified 25 of the 170 DGRs and assigned them as bacterial in origin, while 57 were reported as associated with a prophage or inactive phage remnant (Additional file 2) [6, 8, 10–12, 16]. This analysis identified 74 novel DGRs and expands their observed association with temperate phages across diverse phyla.

**Fig. 1:**
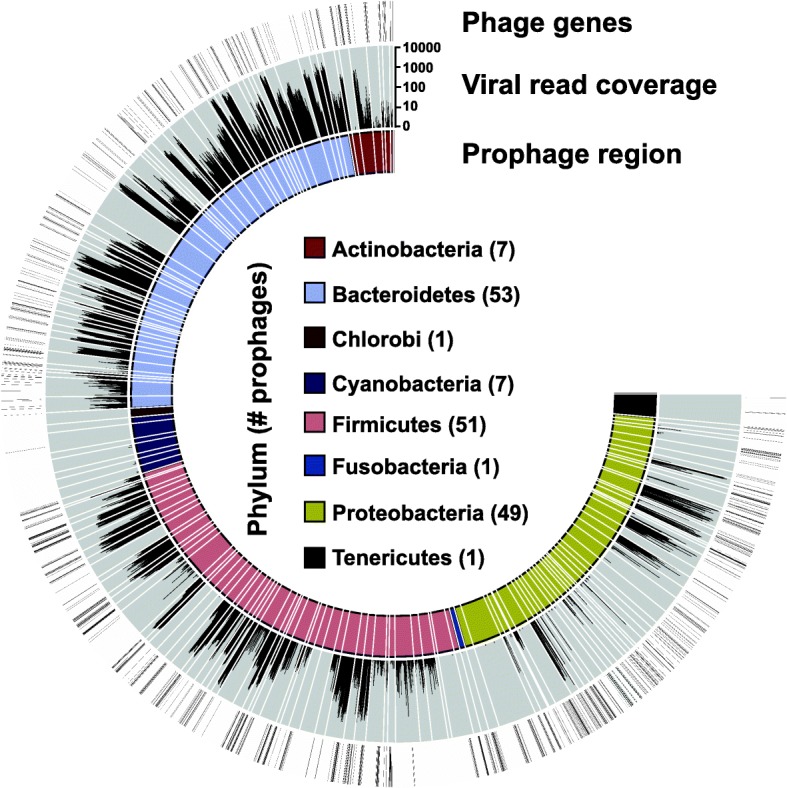
DGRs in bacteria are encoded by functional prophages. Predicted prophage-containing regions with a DGR (*n* = 170) are depicted on the inner ring and colored according to host phylum. Each predicted prophage-containing region was used as a reference to align sequencing reads from human viromes. The coverage plot of reads for each region is shown on the middle ring. ORFs whose annotations contained the keyword “phage” are indicated with a hash mark on the outer ring. Sequences of all predicted prophage-containing regions and their annotations are available in Additional files 11 and 3, respectively

Virions produced by prophages can be captured during viral-particle enrichment steps as part of viral metagenome preparation. Thus, predictions of the PhiSpy algorithm were tested by determining whether viral metagenome reads could be recruited to sequences of all 170 of the predicted prophage regions. Because 104 of the 170 DGR-containing lysogens belonged to the Bacteroidetes or Firmicutes phyla (Additional file 2), whose members dominate the human gastrointestinal tract [17], publicly available viral metagenomes from human skin and fecal samples were downloaded (*n* = 1,366). Approximately three billion quality-filtered sequence reads were concatenated into a single file and aligned to each predicted prophage region (Fig. 1). Ninety-two of the 170 regions recruited viral reads at > 10× mean fold coverage, demonstrating the presence of an integrated phage capable of producing virions (Additional file 2). Predicted prophage regions contained identifiable phage genes, such as capsids, tails, and integrases, validating the recruitment of reads to a temperate phage (Additional file 3). Through the bioinformatic approach presented here, 92 DGRs were classified as viral in origin.

All of the phages possessing a DGR, including BPP-1 and the two *Vibrio* phages, exhibit a temperate lifestyle [4, 18, 19]. To determine if this observation was an artifact of temperate phages being overrepresented in the RefSeq, PhagesDB, and prophage collection, all phage life histories were classified using PHACTS [20]. The majority of phages in these databases could not have a lifestyle assigned (*n* = 2,911) (Additional file 4). Where a lifestyle could be confidently assigned, 1,181 phages were predicted to be temperate and 332 were predicted to be lytic. However, despite the database bias towards temperate phages, the observed number of DGRs in temperate phages was still higher than expected (χ^2^, **P = 0.0012). Therefore, DGRs are preferentially encoded by temperate phages.

### *Bacteroides dorei* is lysogenized by a DGR-containing temperate phage

**Fig. 2:**
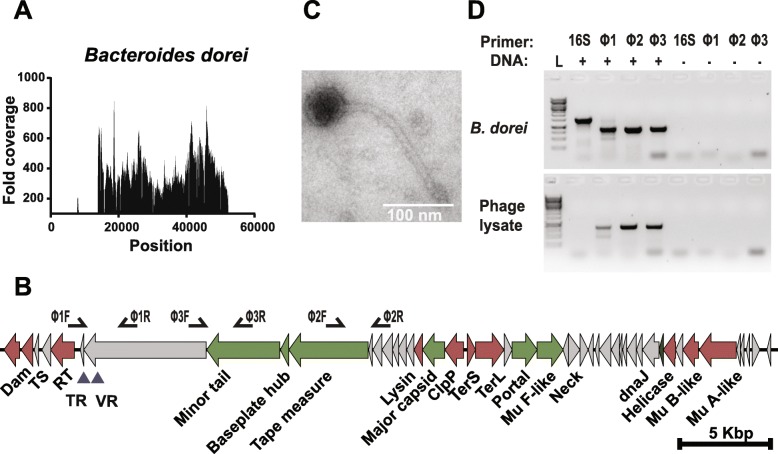
*Bacteroides dorei* is lysogenized by a DGR-containing temperate phage. **a** Coverage plot of viral reads aligning to a 66 kb region in *Bacteroides dorei* predicted to contain a prophage. **b** ORFs encoded by the discrete 43 kb region with high read coverage are represented by arrows and colored according to function. Red, enzymatic; green, structural; gray, hypothetical. The DGR components are indicated as RT, reverse transcriptase; TR, template repeat; VR, variable repeat. **c** Transmission electron microscopy image of a phage lysate prepared from an induced culture of *B. dorei* (scale bar = 100 nm). **d** Agarose gel showing PCR products using primers indicated on **b** and universal 16S rRNA gene primers. The upper panel depicts PCR products using *B. dorei* DNA as a template and the lower panel depicts products from the purified phage lysate. All primer sequences are listed in Additional file 13. For both panels, the four lanes on the right are no-template controls

Several regions predicted to contain a DGR-encoding prophage recruited reads from multiple independent studies (Additional file 5), which was unexpected given the high interpersonal diversity of human viromes [21]. A 66-kb region in *Bacteroides dorei* was further examined because it recruited reads from 13 different human viral studies with a mean fold coverage of 229× (Fig. 2a). The reads aligned to a discrete 43 kb locus encoding 45 ORFs. Seven ORFs with significant homology to known phage structural proteins could be annotated, including two tail proteins, a baseplate, portal, neck, and two capsid genes (Fig. 2b and Additional file 6). Genes required for phage genome packaging and integration were detected, including a large and small terminase and phage Mu-like transposase. The DGR cassette was composed of an RT with two adjacent 117 bp repeats that were mismatched at 14 positions corresponding to adenine bases. Proximal to the RT gene was a small ORF whose amino acid sequence had a similar isoelectric point and molecular weight as the accessory variability determinant of BPP-1 [4]. The VR was located at the C terminus (residues 2188–2225) of a large ORF possessing a C-type lectin domain. Collectively, these features indicated the 43 kb region was a prophage genome that harbored a DGR cassette.

Phage induction assays on a culture of *B. dorei* were performed to validate mature virion production in vitro. A culture of *B. dorei* was obtained and cultured anaerobically at 37 °C. The culture was treated with the antibiotic carbadox, a known prophage inducing agent [22–24]. After 10–12 h of anaerobic growth, bacterial cells were removed. Transmission electron microscopy identified a virus with a 200 nm flexible tail typical of the *Siphoviridae* (Fig. 2c). Lastly, we confirmed the observed phage contained the predicted prophage genotype using PCR (Fig. 2d). This phage was named *Bacteroides dorei* Hankyphage and exemplifies the power of the bioinformatic pipeline to identify inducible prophages, including those that encode DGRs.

### Hankyphage exhibits broad-host-range and global distribution

**Fig. 3:**
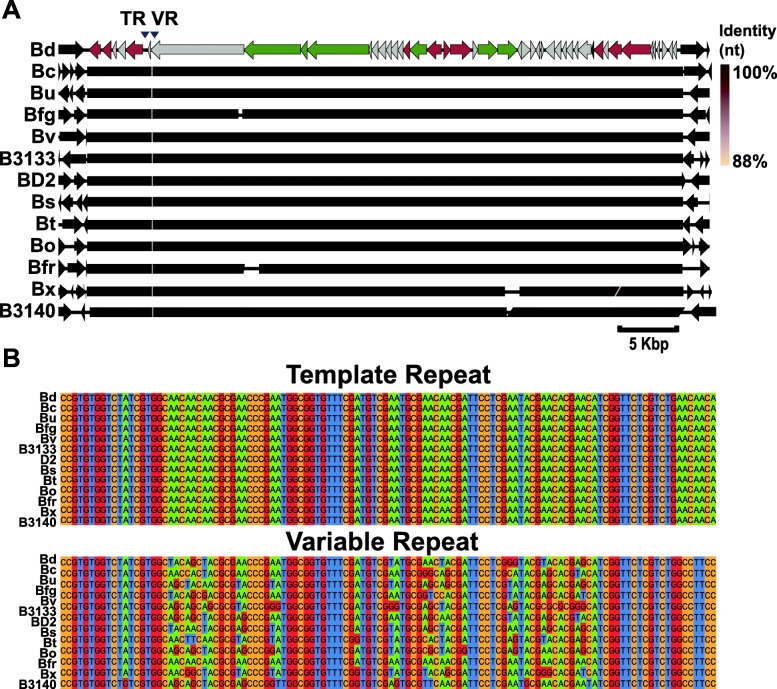
Hankyphage lysogenizes 13 *Bacteroides* species. **a** The Hankyphage genome (top) is color-coded as before and was used to query the NCBI RefSeq database of bacterial genomes. The resulting alignments are color-coded according to the pairwise nucleotide identity. Regions flanking Hankyphage (2 kb) in each subject are depicted as black arrows and produced no significant alignments to each other. The subject sequences are Bc, *B. cellulosilyticus*; Bu, *B. uniformis*; Bfg, *B. finegoldii*; Bv*, B. vulgatus*; B3133, *B. sp*. *3_1_33FAA*; BD2, *B. sp. D2*; Bs*, B. salyersiae*; Bt, *B. thetaiotaomicron*; Bo, *B. ovatus*; Bfr, *B. fragilis*; Bx, *B. xylanisolvens*; and B3140, *B. sp. 3_1_40A.* **b** Alignments of the TRs and VRs from Hankyphage in each *Bacteroides* species are shown. Each nucleotide base is color-coded for visualization of mismatches in the variable repeat

Plaque assays combining a cell-free phage lysate and susceptible bacterial hosts can determine the minimal host range of a phage [25]. A phage lysate was prepared from an induced culture of *B. dorei* and combined with a naïve strain of *Bacteroides dorei* in top agar under anaerobic conditions. Lawns of the naïve strain or additional *Bacteroides* spp. did not yield observable plaques (data not shown). As an alternative, the NCBI RefSeq database of bacterial genomes (accessed 07/2017) was queried for the Hankyphage genome. The complete, 43 kb Hankyphage genome was present in 13 unique *Bacteroides* species at > 95% nucleotide identity (Fig. 3a). Consistent with Mu-like transposition, the site of integration was random and flanked by 5 bp direct repeats [26] (Fig. 3a). Thus, Hankyphage exhibits broad-host-range and each tropic variant was assigned a unique indication (Hankyphage p00–Hankyphage p12).

To test if the DGR cassette diversifies the variable repeat in different *Bacteroides* hosts, the TRs and cognate VRs of each tropic variant were extracted for multiple alignments (Clustal Omega). The aligned TRs contained no substitutions while the VRs exhibited adenine-specific substitutions in asparagine codons, a distinguishing attribute of DGR-mediated variation [4] (Fig. 3b). None of the tropic variants shared the same VR sequence. Interestingly, two tropic variants (*B. dorei* and *B. cellulosilyticus*) only differed in the sequence of their VR along the entirety of their aligned genomes. The VR contained 21 variable positions, enabling Hankyphage to explore a sequence space of potentially 10^12^ unique variants. Despite the similar capacity for sequence variation as the BPP-1 tail fiber, Hankyphage’s DGR target protein shared no sequence similarity with any characterized protein (Additional file 6).

**Fig. 4:**
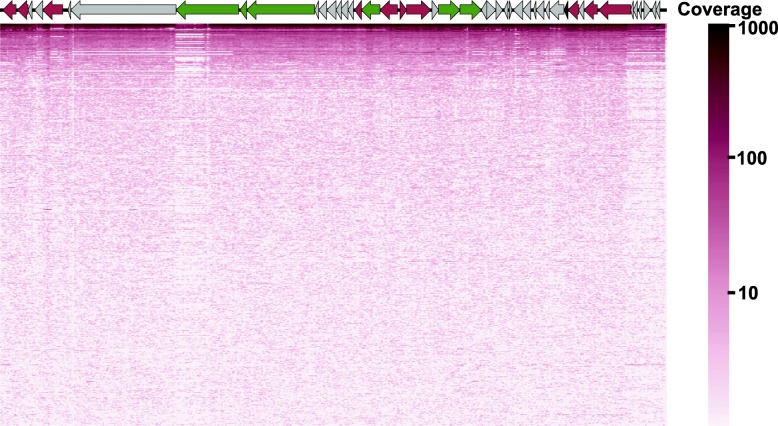
Coverage plot of the Hankyphage genome in metagenomes. Each row is a metagenome (*n* = 500). The x-axis of the heat map displays the 42,831 bp length of the Hankyphage genome. The color bar is scaled proportionally to the coverage of each nucleotide in the Hankyphage genome. Details of the read alignments for all metagenomes are available in Additional file 7

To estimate the ubiquity of Hankyphage in the environment, total-community metagenomes and viromes available on the NCBI SRA were searched for reads matching the Hankyphage genome. An untargeted selection of 37,583 total-community metagenomes from the NCBI SRA (accessed 03/2018) spanning diverse microbial environments was curated previously [27]. In total, 12,774 metagenomes contained > 1 read aligning to the Hankyphage genome (12,774/37,583, 34%) (Fig. 4 and Additional file 7). The same analysis was repeated using the previous database of 1,366 human-associated viromes sampled from geographically separated individuals. Sequencing reads recruited at > 97% nucleotide identity could be recovered from 221 of 1,366 viromes (Additional file 8). All 221 viromes were derived from human fecal samples (221/1038) while none of the human skin virome reads were recruited (0/329). For a given virome sample, Hankyphage was present at 4 × 10^−8^ to 4 × 10^−2^ fractional abundance (Additional file 8). Using these data to estimate the number of Hankyphage virions, approximately 3.3 × 10^17^ exist globally (Additional file 9). In summary, Hankyphage is present in microbial environments, namely the human gastrointestinal tract, from locations sampled around the world.

### Function of hypervariable proteins

To assess the functional role of all the DGR cassettes identified, structures of the variable repeat-containing ORFs were predicted using Phyre2 [28]. Five of the ORFs exhibited structural homology to the major tropism determinant of phage BPP-1, signifying these phages may use a similar strategy as BPP-1 to mutagenize their tail fibers and broaden host range (Additional file 10) [4]. None of the other hypervariable proteins were homologous to known phage proteins; however, 57 hypervariable genes were homologous to a DGR target protein from *Thermus aquaticus* and 29 were homologous to a pilin tip from *Bacteroides ovatus*. The pilin structure was recently shown to contain a C-type lectin domain, supporting previous observations that this fold is a conserved target of DGRs [13, 29]. Overall, the functional roles of these hypervariable proteins remain to be determined.

## Discussion

By combining a prophage prediction algorithm with mapping reads from viromes, 92 phage-encoded DGRs were identified. Many of the proteins targeted for diversification contain a subtype of the C-type lectin fold, suggesting a role in binding interactions with diverse ligands. Phage DGR proteins lack detectible transmembrane regions or signal peptides found on surface-displayed bacterial DGR proteins [13, 30] (Additional file 10). While this supports their phage origin, discerning whether the DGR target proteins are components of functional prophages or defective remnants is difficult. Here, a generalized bioinformatic pipeline was employed to identify functional prophages where viral read data were available.

In one case, an integrated prophage from *B. dorei* was induced, thereby verifying the bioinformatic prediction. This temperate bacteriophage, Hankyphage, is the fifth phage to be induced that possesses a DGR. Hankyphage was detected as a complete prophage in 13 different *Bacteroides* species at > 95% nucleotide identity. Furthermore, Hankyphage’s complete genome was recovered in whole-community and viral metagenomes. These observations suggest that Hankyphage can infect a wide-range of *Bacteroides* in diverse environments despite not infecting naïve *Bacteroides* hosts in vitro. Additional factors may be required for a productive infection outside the gastrointestinal tract. Previous analyses of intestinal viral communities demonstrate few genotypes are shared between individuals [21]. However, analysis of metagenomes generated from human fecal samples collected around the globe discloses that Hankyphage resides in the gastrointestinal tract of approximately 50% of the human population (Additional file 9).

Despite there being hundreds of lytic phage genomes in public databases, DGRs were only identified in temperate phages. Given that the global phage diversity far exceeds that of cultured isolates, identification of a DGR-containing lytic phage may be forthcoming [31]. Nevertheless, DGR-diversification may provide increased functionality for temperate phages. Targeted mutagenesis of phage genes would offset accurate replication by bacterial DNA polymerases, poising temperate phages for dynamic responses to changing environmental conditions. That the majority of DGR-containing phages lysogenize human gut commensals suggests that DGRs are advantageous in host-associated environments. Similarly, DGRs are enriched in host-associated bacteria and archaea with reduced genome sizes, highlighting the parallel emergence of hypervariability cassettes in host-associated environments [10].

The variable repeats of known DGRs lie within C-type lectin folds and Ig-like domains [2, 12]. Both C-type lectins and Ig-like domains are encoded by phages, including Hankyphage and two other *Bacteroides* phages [32–34]. In phage T4, the Ig-like domain-containing protein Hoc mediates phage adherence to mucus, thereby increasing the rate of T4 adsorption onto *E. coli* host cells [35]. Consistent with this bacteriophage adherence to mucus model, Hankyphage may employ a similar strategy. By utilizing a C-type lectin to adhere to mucus, Hankyphage may increase the rate of encounter with *Bacteroides* hosts in mucus. Targeted hypervariation in the C-type lectin could modulate phage diffusion in mucus, enabling optimal search strategies in different mucosal environments.

## Conclusions

In this study, we present evidence that DGR cassettes are encoded by temperate bacteriophages integrated in the chromosomes of bacteria as prophages. Many of these DGRs were previously considered to be bacterial in origin. While the functions of these hypervariable phage proteins remain enigmatic, this research will enable future dissection of their roles in phage life cycles. Moreover, the isolation of a single phage shared amongst an estimated one-half of the human population is an exception to our current understanding that the human viral community is largely composed of unique viruses.

## Methods

### Identification of DGRs

Three databases were searched for DGR cassettes. The first database contained bacteriophage genomes available on the RefSeq database dereplicated at 98% identity using CD-HIT (*n* = 1881, accessed 06/2016) [36, 37]. A second database of actinobacteriophage genomes (*n* = 2322, accessed 12/2017) was downloaded from the PhagesDB website [38]. A third database of 31,946 predicted prophages was generated from 11,278 bacterial and archaeal genomes using the program PhiSpy [15]. All predicted prophage sequences are available from [39]. Sequences in each database were screened for reverse transcriptases (RTs) (Pfam model PF00078) using HMMer [40]. Sequences with RTs were subsequently scanned for template repeats (TRs) and variable repeats (VRs) using the program DiGRef with default settings (minimum 10 adenines in TR, minimum 7 adenine substitutions in VR) modified to accept local inputs [8]. Template repeat and variable repeat alignments were made using Clustal Omega and visualized with JalView [41, 42]. Life history assignments for phages in the databases (RefSeq, PhagesDB, and the DGR-containing predicted prophage regions) were accomplished using PHACTS [20]. A lifestyle assignment was considered confident if the average score of the trees minus the standard deviation was > 0.5. Otherwise, no life history assignment was made.

### Viral read mapping to predicted prophage-containing regions

A collection of human-associated viromes available from the NCBI sequence read archive (SRA) was previously curated [31]. Additional human viromes were added to the database and all accessions are provided (Additional file 8). All of these viromes were downloaded from the SRA using the fastq-dump utility, quality filtered and dereplicated with Prinseq employing the following flags: derep 12345 -lc_method entropy -lc_threshold 50 -trim_qual_left 15 -trim_qual_right 15 -trim_qual_type mean -trim_qual_rule lt -trim_qual_window 2 -trim_tail_left 5 -trim_tail_right 5 -min_len 60 -min_qual_mean 15 -ns_max_p 1 [43]. Reads from each virome were concatenated into a single file and mapped to predicted prophage-containing regions harboring a DGR (*n* = 170, Additional file 11) using Bowtie2 with default parameters [44]. Coverage was calculated with Samtools and visualized using Anvio [45, 46]. To calculate the fractional abundance of *Bacteroides dorei* Hankyphage in each sample, reads from each virome were mapped to the Hankyphage genome at 97% identity using SMALT [47]. The fractional abundance of Hankyphage in each virome was calculated as previously described [31].

### Prophage genome annotation

Open reading frames (ORFs) in predicted prophage-containing regions were called by Prodigal and annotated by BLASTp searches against the NCBI COG database, where hits are considered significant if the *e* value is < 10^−5^ [48, 49]. Additional annotations were determined by HMM searches against the Pfam-A database with a bitscore cutoff of > 25 [50, 51]. ORFs containing a variable repeat were analyzed using Phyre2 and Phobius [28, 52]. To identify the host range of Hankyphage, the phage genome (Additional file 12) was used as a query against all subject *Bacteroides* genomes available on RefSeq using BLASTn (accessed 06/2017). Query-subject alignments were visualized using Easyfig [53].

### Metagenome read mapping to Hankyphage

A collection of whole-community metagenomes available from the NCBI SRA was previously curated [27]. An untargeted selection of 37,583 metagenomes was downloaded using the fastq-dump utility of the SRA toolkit and mapped to the Hankyphage genome using Bowtie2 with default parameters [54–56]. Binary Alignment Map (BAM) files were sorted and indexed using Samtools version 1.7 [46]. Heatmaps were generated from the BAM files using publicly available scripts [57].

### Phage induction, PCR, and transmission electron microscopy

A culture of *Bacteroides dorei* strain CL02T12C06 (HM-719) was obtained through BEI Resources, NIAID, NIH as part of the Human Microbiome Project. *B. dorei* was maintained on supplemented brain heart infusion media (BHIS) [38 g/L BHI, 5 g/L yeast extract, 1.5 g/L L-cysteine, 1 mM CaCl_2_, 10 mM MgSO_4_, 1 μg/L vitamin K3, 0.24 μg/L histidine-hematin]. BHIS broth was inoculated with a glycerol stock of *B. dorei* and cultured anaerobically at 37 °C. Early-log cultures were induced for prophage with 8 μg/mL carbadox and harvested after 8–10 h of growth. The cultures were clarified of bacterial cells through chloroform treatment and syringe-driven 0.22 μm filtration. The clarified supernatant was stored at 4 °C over 0.1 volumes chloroform and used for downstream assays. For PCR, the phage lysate was treated with 3 U of DNase I and incubated for 1 h at 37 °C, followed by heat inactivation for 10 min at 99 °C. Primer sequences are listed in Additional file 13. For transmission electron microscopy, 30 μl of the purified phage lysate was added to glow-discharged 300-mesh copper grids coated with 10 nm formvar and 1 nm carbon (Electron Microscopy Sciences, PA) for 3 min. To remove the salts in the buffer, the grids were rinsed three times by drops of distilled water (20 μl). The grids were then negatively stained with uranyl acetate (0.5%) for 15 s, dried and examined with a FEI Tecnai T12 TEM (FEI, Hillsboro, OR) operating at 120 kV at the SDSU Electron Microscopy Facility. Micrographs were taken by an AMT HX41 side-mounted digital camera (Advanced Microscopy Technique, Woburn, MA).

## Additional files

Additional file 1: DGRs identified in isolated phages from the RefSeq database. For each DGR, annotation of the VR-encoding ORF was made using Phyre2. A domain analysis was conducted using HMMer against the Pfam-A database. Lifestyle assignment was extracted from the literature. (XLSX 9 kb)

Additional file 2: Nucleotide coordinates, taxonomy, and coverage of the DGR cassettes. The nucleotide coordinates of the reverse transcriptase, template repeat and variable repeat on the predicted prophage-containing region are listed in columns A–H (only the first TR/VR pair is reported). Where possible, the nucleotide coordinates of the prophage region in the RefSeq database is listed in columns H–J, along with the taxonomic details of the host organism in columns K–R. The mean coverage of mapped sequencing reads against each predicted prophage is reported in column U. (XLSX 41 kb)

Additional file 3: ORFs in predicted prophage-containing regions. ORFs in all predicted prophage-containing regions were called by Prodigal and assigned a unique id. The nucleotide coordinates and sequence of each ORF is provided. For significant hits, the description and accession of each COG category and Pfam-A family is listed. (XLSX 3960 kb)

Additional file 4: Proportion of temperate and lytic viruses in the phage databases (RefSeq, PhagesDB, and DGR-containing prophage collection). Each phage life history was classified using PHACTS as ‘lytic’ or ‘temperate’ if the trees voting for that lifestyle is > 0.5. Otherwise, the phage lifestyle is listed as ‘N/A’. (XLSX 118 kb)

Additional file 5: Predicted prophage-containing regions with a DGR (*n* = 30) that recruit reads from multiple human virome studies. Each predicted prophage-containing region was used as a reference to align sequencing reads from human viromes. The coverage plot of recruited reads from each study are shown on the middle rings, listed according to the first author of the study (log scale, 0 – 1000× fold coverage). (PDF 2472 kb)

Additional file 6: ORFs encoded in the Hankyphage genome. Functional annotations for each ORF were determined by comparisons to the Pfam-A, Phyre2, or conserved domain database. For significant hits, the database and accession to the hit are listed with the corresponding *e-*value or confidence score. (XLSX 12 kb)

Additional file 7: Metagenomic survey of the SRA for Hankyphage. The Hankyphage genome was used as a reference to align reads from whole-community metagenomes using Bowtie2. The number of aligned reads and percent coverage of the Hankyphage genome is reported. Where possible, the abstract from each study is provided. (XLSX 947 kb)

Additional file 8: Fractional abundance of Hankyphage in viromes. The Hankyphage genome was used as a reference to align reads from each human virome at 97% identity. For each virome containing > 1 aligned read (*n* = 221), the fractional abundance of Hankyphage was calculated [31]. The related metadata for each virome was extracted from the SRA database and listed. (XLSX 78 kb)

Additional file 9: Calculation of the global number of Hankyphage virions. (DOCX 37 kb)

Additional file 10: Annotation of the VR-containing ORFs in DGRs. All ORFs with a VR were annotated using Phyre2 and HMM searches against the Pfam-A database. Annotations were only considered significant if the Phyre2 confidence is > 97 or HMMer bitscore is > 25. Otherwise, the annotation is listed as N/A. The presence or absence of transmembrane-spanning regions and signal peptides were identified using Phobius and indicated as “yes,” “no,” and “signal peptide,” respectively. (XLSX 17 kb)

Additional file 11: Sequences of all 170 predicted prophage-containing regions. (FASTA 11271 kb)

Additional file 12: Sequence of Hankyphage. (GBK 81 kb)

Additional file 13: Primers used in the study (written 5′ to 3′). (DOCX 22 kb)
