# Supplementary material for: A diversity-generating retroelement encoded by a globally ubiquitous Bacteroides phage
Source: Microbiome. 2018 Oct 23;6:191. doi: 10.1186/s40168-018-0573-6 (PMC6199706; doi:10.1186/s40168-018-0573-6)
Supplement: Supplementary file 5 — Predicted prophage-containing regions with a DGR (n = 30) that recruit reads from multiple human virome studies. Each predicted prophage-containing region was used as a reference to align sequencing reads from human viromes. The coverage plot of recruited reads from each study are shown on the middle rings, listed according to the first author of the study (log scale, 0 – 1000× fold coverage). (PDF 2472 kb) [file 40168_2018_573_MOESM5_ESM.pdf]

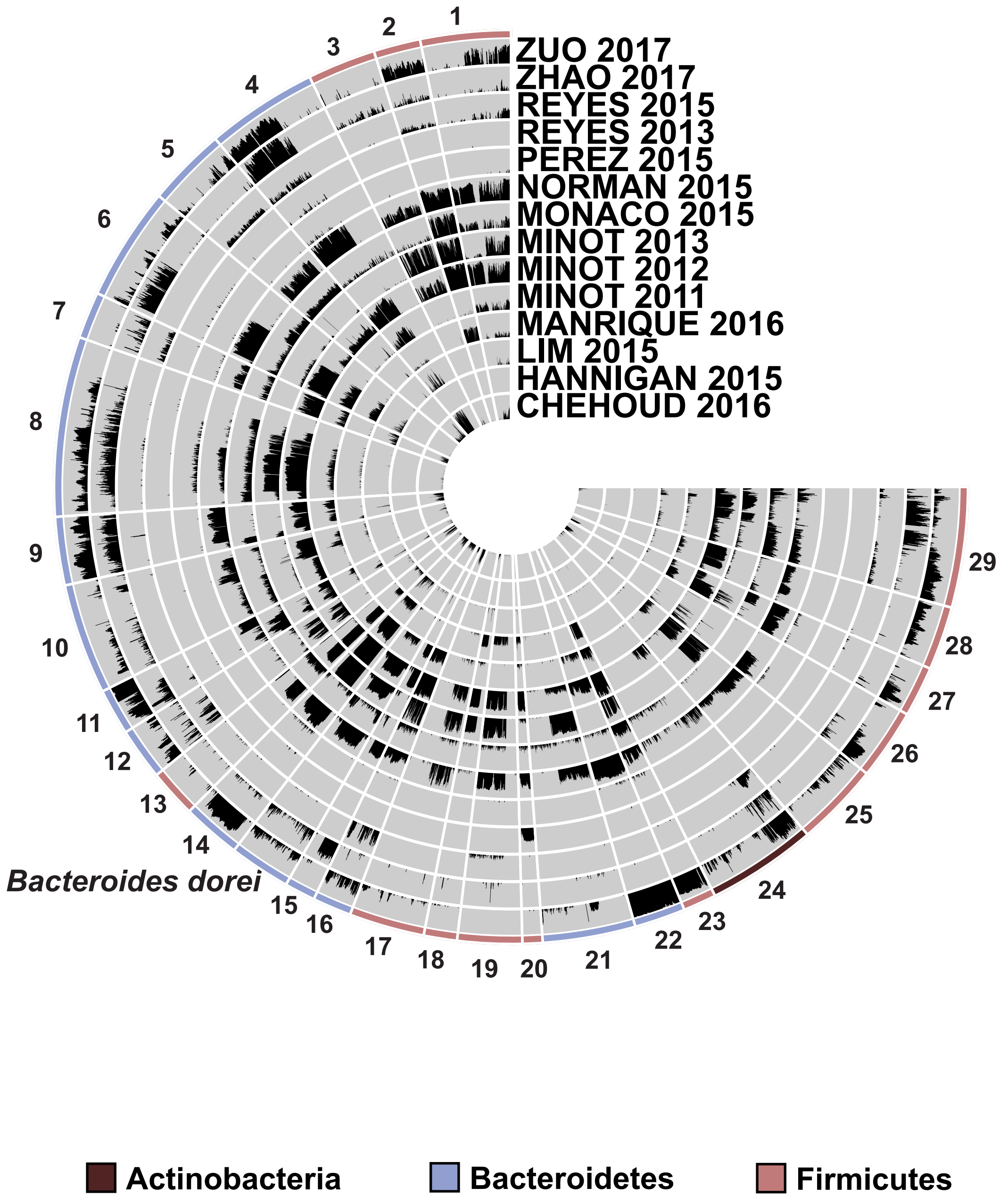

1. g.3125.pp.4	7. g.1648.pp.1	13. g.2141.pp.5	19. g.1375.pp.2	25. g.27096.pp.1
2. g.27137.pp.4	8. g.1601.pp.2	14. g.1750.pp.2	20. g.1363.pp.1	26. g.27096.pp.7
3. g.1613.pp.5	9. g.28925.pp.2	15. g.31350.pp.6	21. g.3099.pp.1	27. g.27096.pp.15
4. g.27474.pp.2	10. g.27099.pp.2	16. g.28920.pp.6	22. g.1522.pp.3	28. g.1372.pp.5
5. g.28920.pp.7	11. g.27343.pp.3	17. g.1380.pp.3	23. g.2234.pp.4	29. g.3105.pp.8
6. g.2542.pp.7	12. g.2867.pp.3	18. g.27128.pp.10	24. g.31087.pp.3	
