# Supplementary material for: A diversity-generating retroelement encoded by a globally ubiquitous Bacteroides phage
Source: Microbiome. 2018 Oct 23;6:191. doi: 10.1186/s40168-018-0573-6 (PMC6199706; doi:10.1186/s40168-018-0573-6)
Supplement: Supplementary file 9 — Calculation of the global number of Hankyphage virions. (DOCX 37 kb) [file 40168_2018_573_MOESM9_ESM.docx]

Additional file 9

**Equation S1:** Calculation of the global number of Hankyphage virions.

$Virions=\phi_{Hanky}\times H\times p\times n$ (1)

Where $\phi_{Hanky}$ is the average fractional abundance of Hankyphage in the human viral community, $H$ equals the number of human associated VLPs, $p$ is the fraction of individuals with Hankyphage and $n$ is the number humans globally.

The average fractional abundance of Hankyphage ($\phi_{Hanky})$ where present in viromes was calculated from additional file 8. The number of human-associated VLPs ($H$) is calculated in Cobian-Guemes et al. [29]. To calculate the fraction of individuals with Hankyphage present ($p$), 1811 metagenomes from 11 SRA projects were analyzed and are listed below. If a metagenome contains reads mapping to > 10% of the Hankyphage genome, Hankyphage is considered present in the metagenome. The world population ($n)$ is estimated by the U.S. Census Bureau as of 2018.

| ***Project*** | ***Metagenomes*** | ***Location*** | ***Fraction of metagenomes with Hankyphage present*** |
| --- | --- | --- | --- |
| SRP075633 | 57 | USA | 0.63 |
| SRP000319 | 6 | USA | 0.67 |
| SRP072561 | 17 | Luxembourg | 0.59 |
| ERP013092 | 24 | Finland | 0.59 |
| ERP005989 | 44 | Denmark | 0.55 |
| ERP007090 | 30 | Sweden | 0.53 |
| SRP067755 | 75 | USA | 0.51 |
| ERP014167 | 8 | China | 0.50 |
| SRP002163 | 1427 | USA | 0.50 |
| ERP015450 | 118 | China | 0.42 |
| SRP056480 | 5 | Tanzania | 0.40 |
| Average |  | | 0.53 |

Solving for each variable, this leads to

$Virions=2.2\times{10}^{-5}\cdot3.19\times{10}^{12}\cdot0.53\cdot{7.4\times10}^{9} = 3.3\times{10}^{17}$ (2)
