# Supplementary material for: A diversity-generating retroelement encoded by a globally ubiquitous Bacteroides phage
Source: Microbiome. 2018 Oct 23;6:191. doi: 10.1186/s40168-018-0573-6 (PMC6199706; doi:10.1186/s40168-018-0573-6)
Supplement: Supplementary file 13 — Primers used in the study (written 5′ to 3′). (DOCX 22 kb) [file 40168_2018_573_MOESM13_ESM.docx]

Additional file 11

Primers used in the study (written 5’ to 3’).

| ***Primer name*** | ***Sequence*** |
| --- | --- |
| Φ1F | 5’GCCTCCTCAATCAACCCTAATG |
| Φ1R | 5’ACCGATGTTCGTGTTCGTATTC |
| Φ2F | 5’ ATTCACCGAAGACAGTACCC |
| Φ2R | 5’ GAAGGAATACGGAAAGATTACGC |
| Φ3F | 5’ GGAGGAAAGGACATAATAACCG |
| Φ3R | 5’CAACTACCTTTACTGGTACGG |
| 16S (27F) | 5’AGAGTTTGATCTGGCTCAG |
| 16S (1492R) | 5’ GGTTACCTTGTTACGACTT |
